# Excess Hydrocortisone Hampers Placental Nutrient Uptake Disrupting Cellular Metabolism

**DOI:** 10.1155/2018/5106174

**Published:** 2018-10-09

**Authors:** Rosa María Mateos, Gema Jiménez, Carmen Álvarez-Gil, Francisco Visiedo, Fátima Rivera-Rodríguez, Celeste Santos-Rosendo, Antonia Rodriguez-Pareja, Germán Perdomo, Alfonso M. Lechuga-Sancho

**Affiliations:** ^1^Department of Biomedicine, Biotechnology and Public Health, University of Cádiz, Cádiz, Spain; ^2^Research Unit, Puerta del Mar University Hospital, Cádiz, Spain; ^3^Department of Obstetrics and Gynecology, Hospital de Jerez, Cádiz, Spain; ^4^Department of Mother and Child Health and Radiology, Pediatric Endocrinology, Puerta del Mar University Hospital, University of Cádiz, Cádiz, Spain; ^5^Universidad de Burgos, Burgos, Spain

## Abstract

Low birth weight increases neonatal morbidity and mortality, and surviving infants have increased risk of metabolic and cardiovascular disturbances later in life, as well as other neurological, psychiatric, and immune complications. A gestational excess of glucocorticoids (GCs) is a well-known cause for fetal growth retardation, but the biological basis for this association remains elusive. Placental growth is closely related to fetal growth. The placenta is the main regulator of nutrient transport to the fetus, resulting from the difference between placental nutrient uptake and the placenta's own metabolism. The aim of this study was to analyze how excess hydrocortisone affects placental glucose and lipid metabolism. Human placenta explants from term physiological pregnancies were cultured for 18 hours under different hydrocortisone concentrations (2.75, 5.5, and 55 mM; 1, 2, and 20 mg/ml). Placental glucose and lipid uptake and the metabolic partitioning of fatty acids were quantified by isotopic techniques, and expression of specific glucose transporter GLUT1 was quantified by western blot. Cell viability was assessed by MTT, immunohistochemistry and caspase activity. We found that excess hydrocortisone impairs glucose uptake and lipoprotein lipase (LPL) activity, coincident with a GC-dose dependent inhibition of fatty acid oxidation and esterification. None of the experimental conditions showed an increased cell death. In conclusion, our results show that GC overexposure exerts a dysfunctional effect on lipid transport and metabolism and glucose uptake in human placental explants. These findings could well be directly related to a reduced placental growth and possibly to a reduced supply of nutrients to the fetus and the consequent fetal growth retardation and metabolic programming.

## 1. Introduction

Low birth weight increases neonatal morbidity and mortality, and surviving infants have higher risk of cardiovascular, metabolic, neuroendocrine, and psychiatric disorders later in life. These increased risks are hypothesized to be the consequence of a fetal adaptive response to chronic nutrient shortage during gestation, to ensure the development of insulin independent organs such as central nervous system, and may permanently affect adult health [1, 2].

Nutrient availability determines fetal growth [3]. The placenta's ability to supply nutrients to the fetus from the mother depends on several factors such as maternal nutrition status, uterine vascularization, placental size and morphology, umbilical blood flow, the maternal-fetal concentration gradient, the density and activity of placental specific nutrient transporters, and placental metabolism [4–6]. Placental dysfunction may restrict fetal growth by reducing fetal nutrient supply, inducing intrauterine growth restriction (IUGR), and fetal adaptive responses [7–9].

Placental weight, as a marker of the surface area available for nutrient exchange, is a major determinant of fetal growth and birth weight. A positive correlation between placental and fetal weight at term has been well documented [10, 11]. Indeed, epidemiological studies suggest that placental weight might be an independent marker of the long-term health outcome of the fetus [12]. In general, low placental weight at term is associated with lower neonatal weight and predicts hypertension and coronary disease in offspring [13].

The placenta's capacity for nutrient uptake for both, its own metabolic needs and transport to the fetus, can be affected by modifications in the number, density, and distribution of nutrient-specific transporters [14–17]. Physiological levels of glucocorticoids (GCs) are essential for embryo implantation, fetal growth, tissue development, and maturation of various organs to prepare the fetus for the extrauterine life. However, supraphysiological levels of GCs are a well-known cause of fetal growth retardation [18–22] and long term consequences such as persistent hyperactivation of hypothalamus-pituitary-adrenal axis, adult hypertension, hyperglycaemia, and various neurodevelopment alterations [23–25].

There is a clear relationship between elevated GC exposure and placental growth restriction and functional modifications in various species including humans [26]. Although it is known that placental growth restriction is related to the type of GC, the dose, and the therapy duration and timing [27], mechanisms by which GCs impact placental growth remain elusive.

Interestingly, experimental data with ewes revealed that while repeated maternal doses of corticosteroids result in reduced placental and neonatal weights, their direct fetal injection does not affect neither of them [28], suggesting that GCs' effects on neonatal weight are mediated by their repercussion on placental development and function. Indeed, the effects of GCs on placental cell cycle, apoptosis, placental angiogenesis, and nutrient transporters have been extensively studied in different in vitro and in vivo models [26, 29–33]. However, little is known on how GCs (especially hydrocortisone) affect primarily placenta's metabolism, and since they exert key tissue-specific metabolic effects, we aimed in this study to analyze specifically hydrocortisone's impact on placenta's glucose and lipid metabolism.

## 2. Materials and Methods

### 2.1. Study Samples

Placentas were obtained from 9 women with physiological, uneventful, full-term pregnancies, with adequate birth weight newborns at the Obstetrics Department of University Hospital Puerta del Mar in Cádiz, Spain (HUPM, Cádiz) (Table 1). None of the mothers recruited for this study had a history of chronic diseases, hypertension, preeclampsia, infections, or fetal anomalies. All women had uncomplicated singleton pregnancies, delivered by programmed caesarean section without labor, and agreed to participate in the study by written informed consent.

Placentas were collected within 10 minutes following delivery. Each placenta was then weighed and sampled in placental explants (~100 mg wet weight) from the villous placenta region. Each explant was cultured in 24-well dishes containing 2 ml of culture medium per well (RPMI-1640 supplemented with 5 mM glucose, 10% FBS (v/v), 100 units/ml penicillin G, and 100 *μ*g/ml streptomycin) as described previously [34] and hydrocortisone (Actocortina®, Nycomed Pharma) at concentrations of 2.75, 5.5, and 55 mM (1, 2, and 20 mg/ml, respectively), for 18 hours. These hydrocortisone concentrations were set after preliminary experiments on cellular apoptosis and lipid metabolism with increasing hydrocortisone concentrations. We finally set the experiments with a range of doses from the minimal concentration found to induce changes in lipid metabolism, to the highest dose inducing such changes without inducing cell death, as a positive control. Similarly, the timing for incubation was set at 18 hours (the minimum to find changes in lipid metabolism in preliminary time-curve tests). Every experiment was repeated with explants from 4–6 different placentas (n = 4–6).

The study was approved by the Research Ethics Committee of the Puerta del Mar University Hospital and the Cadiz's Bay-La Janda District and performed according to the Declaration of Helsinki.

### 2.2. Materials

Cell culture reagents (RPMI-1640 medium without glucose and fetal bovine serum) were purchased from Gibco, California, USA. The [9,10-^3^H]-palmitic acid, [^3^H]-H_2_O, 2-[1,2-^3^H]-deoxy-D-glucose, [1-^14^C]-mannitol, and liquid scintillation counting cocktail were from Perkin Elmer, Massachusetts, USA. Hydrocortisone sodium phosphate, as GC treatment used in this study, was purchased from Nycomed Pharma (Zurich, Switzerland). Lipoprotein lipase (LPL) was purchased from Sigma-Aldrich (St. Louis, USA). MTT for the colorimetric assay for the nonradioactive quantification of cellular metabolic activity was purchased from Roche (Mannheim, Germany). Apoptosis in the placental explants was analyzed by the Dead-End TM Fluorometric TUNEL System Kit (Promega, Madison, WI, USA) and the Caspase-GLO 3/7 Assay Kit (Promega, Madison, USA).

### 2.3. Glucose Transport Assay

Uptake of [^3^H]-2-DOG was performed* ex vivo* in placental explants, as described previously [35], with the following modifications. Freshly isolated placental explants were preincubated for 18 h in culture medium in the presence or absence of GCs. Afterwards, explants were washed in transport solution buffer (20 mM Hepes-Na pH 7.4, 140 mM NaCl, 5 mM KCl, 2.5 mM MgSO_4_, and 1 M CaCl_2_) at room temperature and immediately transferred to transport solution plus 10 *μ*M 2-DOG (18500 Bq/mL [^3^H]-2-DOG) and 39 mM mannitol (11840 Bq/mL [^14^C]-mannitol) with or without GCs for 1 min. Explants were then removed rapidly, rinsed with cold 0,9% NaCl to stop reactions, blotted, digested in 1 M NaOH, and analyzed for ^14^C and ^3^H content. Glucose transport was defined as nmol of 2-DOG per mg of protein per minute.

### 2.4. Western Blots for GLUT1, Cleaved Caspase 3, and Phospho ERK Protein Levels

Mock and GCs-treated placental explants were dissected and washed with ice-cold PBS, followed by homogenization in lysis buffer (20 mM Tris-HCl pH 7.5, 150 mM NaCl, 1 mM EDTA, 1 mM EGTA, 1% (v/v) Triton X-100, 2.5 mM sodium pyrophosphate, 1 mM *β*-glycerophosphate, 1 mM Na_3_VO_4_, 1 *μ*g/ml leupeptin, and 1 mM phenylmethylsulfonyl fluoride) and protease inhibitor cocktail (Protease Inhibitor Cocktail, Sigma, St. Louis, MO). After 10 min. on ice, extracts were sonicated and centrifuged at 18,000 X g for 10 minutes at 4°C. Pellets were discarded and solubilized proteins (60 *μ*g/sample) were resolved by 10% SDS-PAGE and electrotransferred onto polyvinylidene difluoride (PVDF) filters for immunoblotting by conventional means. Membranes were blocked in 5% skimmed milk at room temperature for 1 hour and then incubated with primary antibody [glucose transporter 1 (Glut-1; 1:2000, Abcam, Cambridge, UK), Cleaved Caspase 3 (Casp-3; 1:1000, Cell Signaling, EEUU), (phospho-)p44/p42 MAP Kinase Antibody (p-Erk1/p-Erk2 and Total Erk1/Erk2; 1:1000, Cell Signaling, EEUU), and *β*-Actin (1:5000, Abcam, Cambridge, UK)] in 5% BSA in PBS at 4°C overnight. After washing off excess primary antibody three times with PBS for 15 minutes, membranes were then incubated with secondary antibody (Anti-Rabbit Peroxidase; 1:2000, Sigma-Aldrich, EEUU). Signals were detected by chemiluminescence (Immun-Start western chemiluminescence kit, Bio-Rad, Madrid, Spain), and band densitometry was quantified with the Image-J software (NIH, USA).

### 2.5. RNA Isolation and Real-Time PCR

For Total RNA isolation we used the RNAspin Mini Kit (GE Healthcare, Buckinghamshire, UK). Reverse transcription was performed with the Transcriptor First Strand cDNA Synthesis Kit (Roche, Mannheim, Germany) using 1 *μ*g of RNA in 20 *μ*l of reaction volume. The cDNA was diluted into 200 *μ*l for later use in real-time PCR. Two primer pairs were used to amplify the human target genes, GLUT-1 and GLUT-3: hGLUT1-F, 5′-GCGGAATTCAATGCTGATGAT-3′; hGLUT1-R, 5′-CAGTTTCGA GAAGCCCATGAG-3′; hGLUT3-F, 5′-TTATCTTCACCGGCTTCCTCA; hGLUT3-R, CAGCATTCAGAAGCGTCCTGGGTTC. *β*-Actin mRNA expression was measured as housekeeping gene with the primers hBACT-F, 5′-TACCACTGGCAT CGT GATGGACT-3′; hBACT-R, 5′-CGTCACACTTCATGATGGAG-3′. No significant differences were detected in the *β*-actin mRNA expression among the samples studied (data not shown). Real-time PCR was carried out in 10 *μ*l of reaction containing 5 *μ*l of SensiFAST™ SYBR No-ROX Kit (Bioline, London, UK), 0.5 *μ*l forward primer, 0.5 *μ*l reverse primer, 3 *μ*l DEPC H_2_O, and 1 *μ*l cDNA template. Following the initial denaturization at 95°C for 3 min, 40 cycles of amplification were performed with the following conditions: incubation at 95°C for 15 s, annealing at 60°C for 20 s, and extension at 72°C for 20 s. The threshold cycles of each mRNA species were determined in triplicate with the use of a Rotor-Gene 6000 System (Corbett, Mortlake, Australia). The mRNA levels of the target genes were expressed as relative folds over those of BACT that was used as the internal reference.

### 2.6. Fatty Acid Oxidation Assay

A stock of fatty acid solution was prepared by conjugating palmitate with essentially fatty acid-free bovine serum albumin (BSA) to generate a solution of 25% (wt/vol) BSA, with 4 mM palmitate for the culture medium, as described previously [34]. Mitochondrial fatty acid oxidation (FAO) assays were performed in placental explants as previously described [34, 36]. Briefly, freshly isolated explants were incubated in culture media in the presence or absence of GCs, plus 1.25% BSA, 0.1 mM cold palmitate, and 18500 Bq/ml [3H]-palmitate at 37°C for 18 h. At the end of the incubation period, the medium was collected and tritiated water determined by the vapor-phase equilibration method of Hughes et al. [37]. FAO was defined as nmol of palmitate per mg of tissue per hour.

### 2.7. Esterification into Total Lipids Assay

The esterification rate in placental explants was determined as previously described [34, 38]. Briefly, after similar incubation conditions to those used for measurements of *β*-oxidation, in the presence or absence of GCs, plus 0.1 mM cold palmitate and 18500 Bq/mL [^3^H]-palmitate at 37° for 18 h., explants were washed 3 times with 2 mL of ice-cold PBS and homogenized in 500 *μ*L of PBS. An aliquot of 100 *μ*L was used to extract the lipid content from samples according to Bligh and Dyer [39]. Afterwards, the radioactive content was determined by liquid scintillation counting. Esterification was defined as nmol of palmitate per mg of tissue per hour.

### 2.8. LPL Activity Assay

Lipoprotein lipase (LPL) mediates the hydrolysis of triglycerides from maternal lipoproteins to obtain free fatty acids. It represents one potential mechanism for increasing placental lipid transport. The LPL activity assay kit is a fluorometric assay which includes a nonfluorescent substrate emulsion that becomes intensely fluorescent upon interaction with LPL. The fluorescence signal is proportional to the LPL activity and can be monitored in a fluorometer (*λ*ex=370 / *λ*em=450nm) following the manufacturer's instructions. LPL activity was defined as nmol of label per mg of protein.

### 2.9. Cellular Metabolic Activity Assay

Cellular metabolic activity of the explants after exposure to GCs was assessed by MTT assay [40]. Placental explants were seeded in 24-well dishes and the Cell Proliferation Kit I (Roche, Mannheim, Germany) was used following the manufacturer's instructions.

### 2.10. Assessment of Placental Apoptosis

#### 2.10.1. TUNEL Assay

Apoptosis was assessed with a commercially available fluorescent terminal deoxynucleotidyl transferase biotin-dUTP nick end labeling (TUNEL) kit (Dead-End TM Fluorometric TUNEL System Kit; Promega, Madison, WI, USA). After being stained according to the manufacturer's instructions, the sections were mounted in Vectashield with DAPI (Vector Laboratories, Vulingame, CA, USA). Three explants from 6 different placentas were included in each experimental condition; three sections per explant were examined, analyzing 10 images from each section, to determine the percentage of TUNEL-positive nuclei.

#### 2.10.2. Caspase Glo Activity Assay

Placental explants lysates were subjected to caspase 3/7 activities measurement with Caspase-Glo 3/7 Assay Kit (Promega, Madison USA). 100 *μ*l of placental lysates (2 *μ*g/*μ*l proteins) plus 100 *μ*l of Caspase-Glo reagent was added to each well and the content of the well was gently mixed with a plate shaker at 300–500 rpm for 30 seconds. The plate was then incubated in darkness at room temperature for 1 hour. The luminescence of each sample was measured in a plate-reading luminometer (Thermo Labsystems). Data of total caspase 3/7 activity in arbitrary units (A.U.) were normalized to the mock sample and by normalizing relative light units (raw data) for the protein content of the lysate.

#### 2.10.3. Cleaved Caspase 3 Determination

We measured levels of cleaved caspase 3 by western blot as described above.

### 2.11. Statistical Analysis

Statistical analysis of data was performed using the SPSS software and Graph Pad Prism 5. The distributions were evaluated using histograms and the Kolmogorov-Smirnov test and data were presented as mean ± standard deviation. For quantitative variables, the descriptive results were expressed by central and dispersion tendency measures (mean, median, and standard deviation). The qualitative variables were expressed by means of frequencies and percentages. Comparisons between more than two groups were performed by Kruskal-Wallis nonparametric test followed by Dunn's test. Pairwise comparisons were performed using Mann-Whitney U test with Bonferroni correction (number of comparisons = 6). P<0.05 was considered for statistical significance.

## 3. Results

### 3.1. Hydrocortisone Decreases Glucose Uptake by Placental Explants, in spite of Increased GLUT1 Protein Levels and GLUT3 mRNA Expression

**Figure 1:**
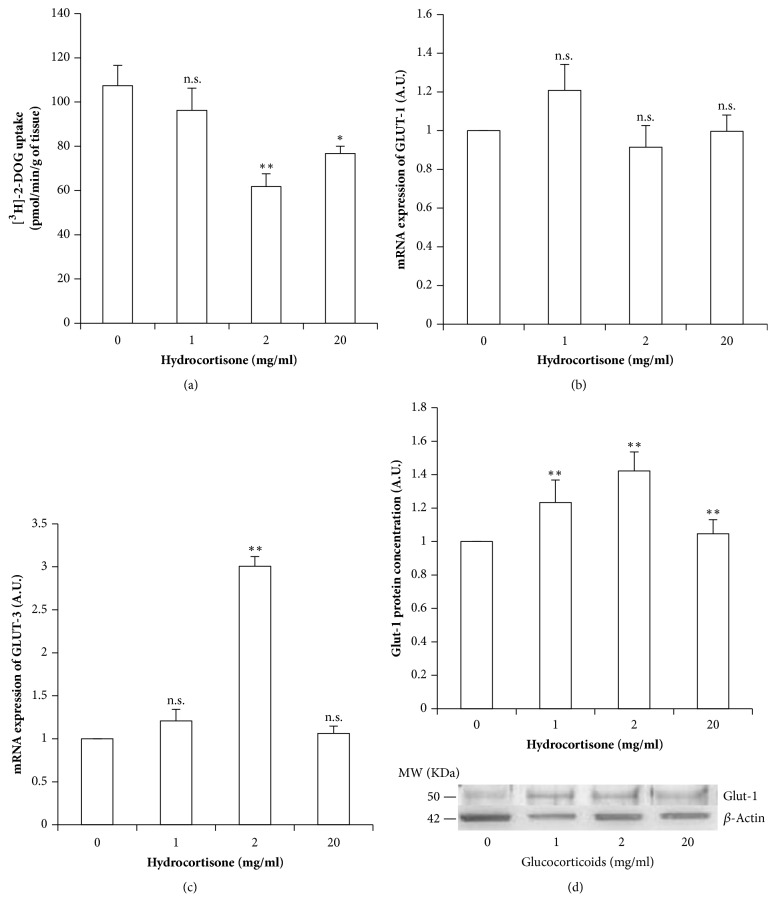
**Hydrocortisone's effects on glucose uptake and glucose transporters in placental explants**. (a) Glucose uptake as measured by [^3^H]-2-DOG incorporation. (b) GLUT1 mRNA expression normalized by actin mRNA expression. (c) GLUT3 mRNA expression normalized by actin mRNA. (d) GLUT1 protein levels as measured by western blot, normalized by actin levels, together with a representative photograph. Values are represented as means ± SEM for 5 independent experiments. *∗*p<0.05; *∗∗*p<0.025; *∗∗∗*p<0.01 relative to control (A.U.: arbitrary units).

When cultured with 1 mg/ml (2.75 mM) of hydrocortisone, there was no change in [^3^H]-2-DOG uptake by explants. However, increasing concentrations of GCs resulted in a ~30-40% decrease in [^3^H]-2-DOG uptake (p <0.05), with respect to 1 mg/ml of GC and control (Figure 1(a)). To analyze if this decrease in glucose uptake could be explained by a decrease in glucose transporters expression or protein levels, we performed real-time PCR to analyze GLUT1 and GLUT3 mRNA expression and quantified GLUT1 protein content by western blot. GC exposure did not induce any significant change in GLUT1 mRNA expression levels in any of the experimental GCs concentrations (Figure 1(b)), but they increased GLUT3 expression at 2 mg/ml (5.5 mM) (Figure 1(c)), in a mirrored image of their effect on glucose uptake. On the contrary, hydrocortisone induced a mild but significant increase in GLUT1 protein level (p=0.007) (Figure 1(d)).

### 3.2. Placenta's Fatty Acid Oxidation and Esterification Are Decreased by Hydrocortisone in a Dose-Dependent Manner

**Figure 2:**
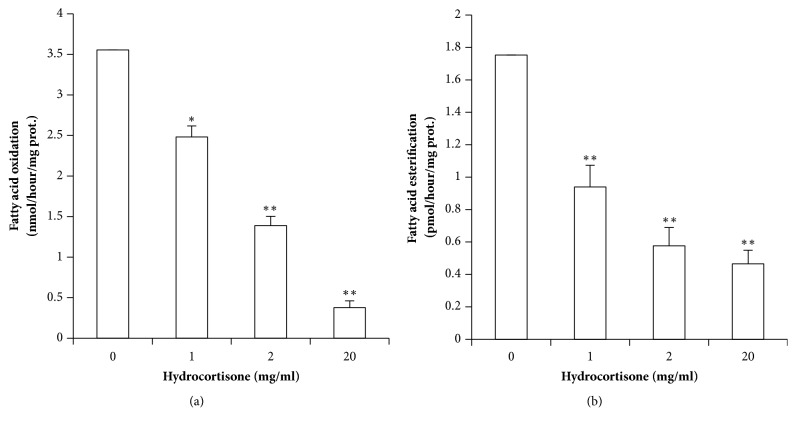
**Hydrocortisone's effects in placental explants' lipid metabolism.** (a) Fatty acid oxidation, in nmol/hour/mg of protein, was inhibited by glucocorticoids in a dose dependent manner. (b) Fatty acid esterification, in pmol/hour/mg of protein, was also decreased in a dose dependent manner. Values are represented as means ± SEM for at least 5 independent experiments, in triplicate. *∗*p≤0.05; *∗∗*p≤0.01; *∗∗∗*p≤0.001 relative to control.

We determined the fatty acid oxidation (FAO) and the fatty acid esterification (FAE) rates of the placental explants cultured with GCs for 18 hours. FAO rate was reduced by ~25%, ~50%, and ~75% in placental explants treated with 2.75, 5.5, and 55 mM (1, 2, and 20 mg/ml), respectively (p=0.05; p=0.008; p=0.01) (Figure 2(a)). Coinciding with reduced FAO activity, FAE in the GC-treated group was also significantly lower compared with control (p<0.01) (Figure 2(b)). Significant differences in FAE were detected already with 2.75 mM (1 mg/ml) of GCs.

### 3.3. LPL Activity Decreased in Placental Explants Treated with Excess of Hydrocortisone

**Figure 3:**
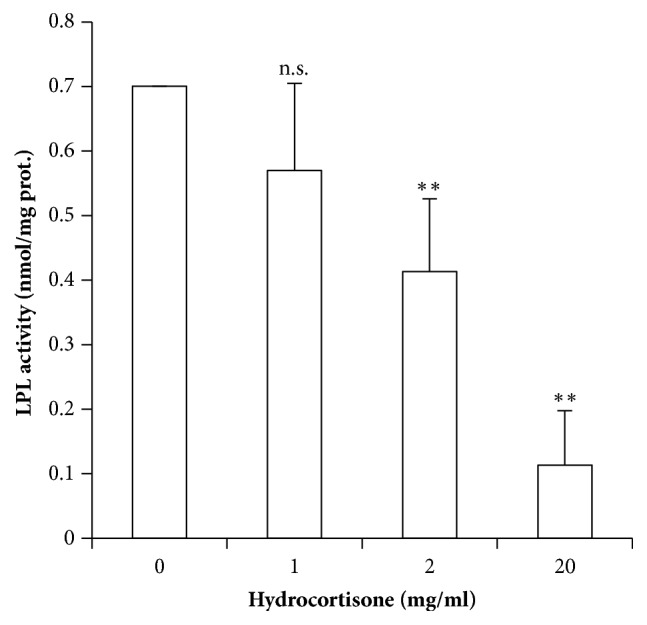
**LPL activity of placental explants with increasing hydrocortisone concentrations.** Values measured in nmol/mg of proteins are represented as means ± SEM for at least 4 independent experiments in triplicate. *∗*p≤0.05; *∗∗*p≤0.01; *∗∗∗*p≤0.001 relative to control.

GCs induced a dose-dependent inhibition of LPL activity in the explants after 18 hours of incubation (Figure 3). GCs at 1 mg/ml (2.75 mM) did not significantly affect LPL activity, but hydrocortisone at 2 and 20 mg/ml (5.5 and 55 mM) induced a ~40% and ~80% decrease in LPL activity, respectively (p<0.01; p<0.001).

### 3.4. Hydrocortisone Decreases Cellular Metabolic Activity in Placental Explants, While Apoptosis Markers Remain Unaltered

**Figure 4:**
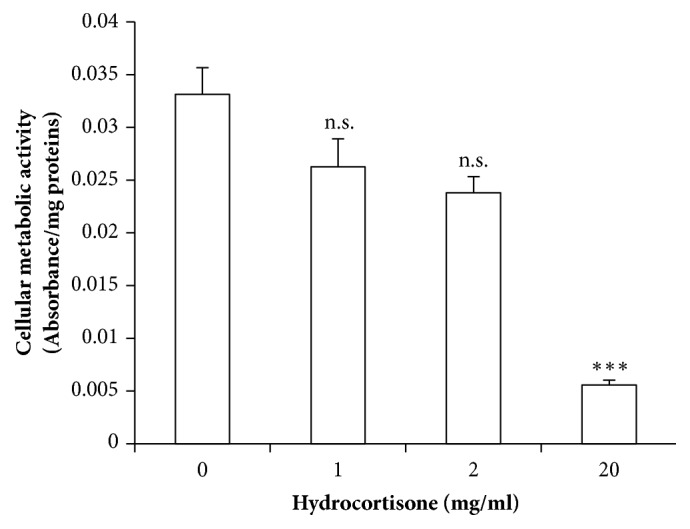
**Mitochondrial activity as measured by MTT. **Absorbance per mg of protein content, represented as means ± SEM for 6 independent experiments. *∗*p<0.05; *∗∗*p<0.025; *∗∗∗*p<0.01 relative to control.

Cellular metabolic activity as measured by MTT assay was decreased after incubation with hydrocortisone, and this decrease became significant with a hydrocortisone concentration of 20 mg/ml (55 mM) (Figure 4). To confirm if the decrease observed in metabolic activity could be explained by apoptosis, three different apoptosis markers were analyzed,* TUNEL* (by immunofluorescence microscopy), total cleaved caspases 3 and 7 (by the Caspase Glo kit; Promega), and finally* cleaved caspase 3* (by western blot).

**Figure 5:**
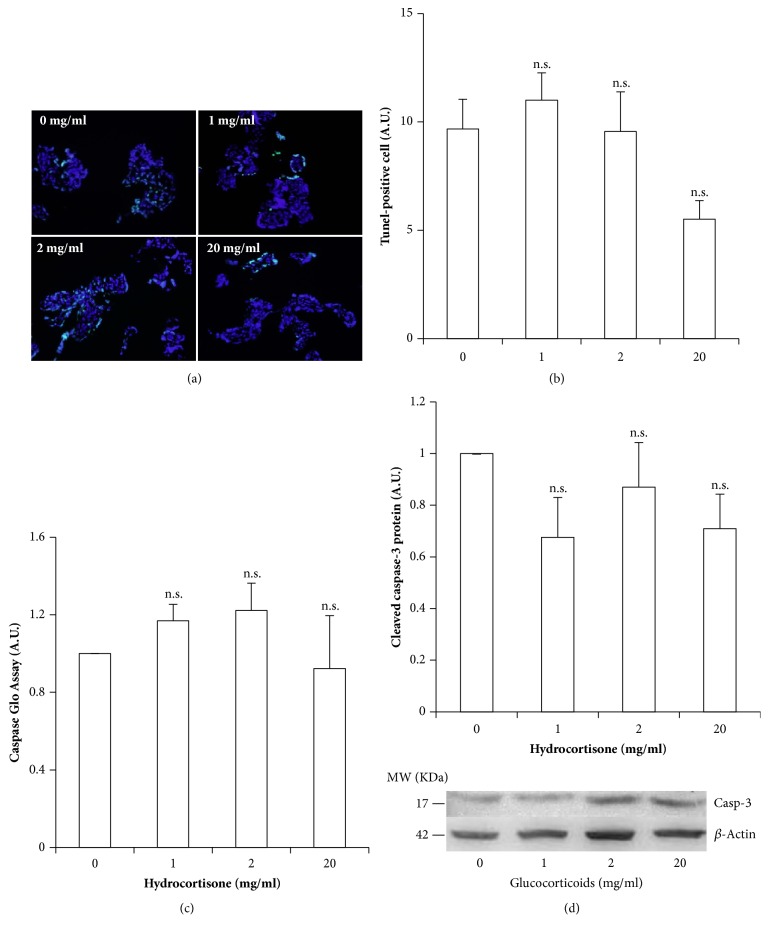
**Assessment of hydrocortisone-induced cellular death by apoptosis in healthy-term placental explants. **(a) Representative microphotographs of TUNEL assay to determine apoptosis in each experimental condition; cells undergoing apoptosis present fluorescent green nuclei. (b) Quantification of the % of TUNEL positive cells per condition. (c) Active caspases 3 and 7 as measured by luminometry, using the Caspase Glo Assay. (d) Cleaved caspase 3 as determined by western blot and normalized by actin levels. Values are presented as means ± SEM for 5 independent experiments. *∗*p≤0.05; *∗∗*p≤0.01; *∗∗∗*p≤0.001 relative to control.

Firstly, a time-concentration curve was carried out to analyze if apoptosis activation was triggered along the 18 hours of the experimental process, being measured from 0-20 mg/ml hydrocortisone at 0, 1, 3, 6, and 18 hours of incubation. No significant differences were detected in any of the apoptosis markers along the experimental conditions assessed in the time-concentration curve (results not shown). We found no significant differences between the control condition and none of the GC concentration in density of TUNEL positive cells (Figures 5(a) and 5(b)), caspase glo (Figure 5(c)), or cleaved caspase 3 concentration (Figure 5(d)).

### 3.5. ERK Phosphorylation Is Decreased by Excess Hydrocortisone

**Figure 6:**
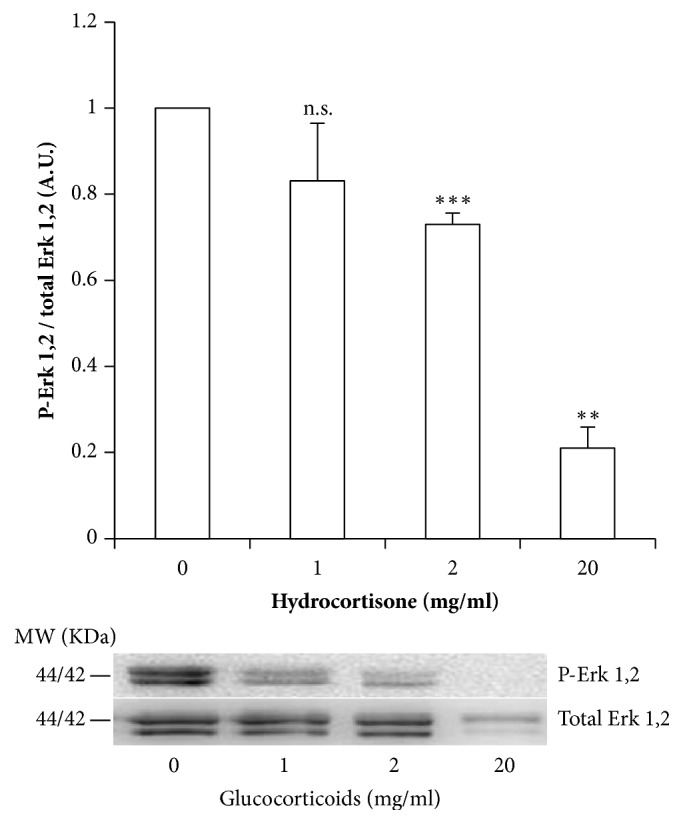
**Dose dependent inhibition of MAPK-signaling pathway in placental explants by hydrocortisone. **Western blot analysis of P-Erk1,2 vs. Total Erk1,2. Values are means ± SEM for 6 independent experiments. *∗*p<0.05; *∗∗*p<0.025; *∗∗∗*p<0.010 relative to control.

We found a dose-dependent decrease of ERK1,2 phosphorylation by GCs. While 1 mg/ml (2.75 mM) of hydrocortisone did not significantly decrease ERK phosphorylation, 2 mg/ml (5.5 mM) induced a ~25% decrease (p<0.001) and 20 mg/ml (55 mM) a ~75% decrease (p<0.01) in phosphorylation (Figure 6).

## 4. Discussion

The placenta is a GC target organ expressing multiple GC receptor isoforms [41]. GCs play a profound impact on cellular metabolism, suppressing cellular glucose uptake, and glycogen synthesis, and also reducing LPL activity together with lipolysis promotion in peripheral tissues, while activating lipogenic pathways in central tissues, favoring peripheral depletion with central depot [42].

We found that GCs excess induced an increase in GLUT1 protein levels, in accordance with data from placental endothelial cells (HPECs) [31, 32] and pregnant rats under dexamethasone treatment [43]. GLUT1 is the only transporter present as a functional protein in the syncytium near term [44]; thus changes in its density and/or activity would deeply influence placental and fetal development. Besides the increase in GLUT1 protein levels, we did not find an increase in its mRNA expression. This may be explained by the finding that inhibitors of mitochondrial activity decrease GLUT1 transcript degradation [45], and we found, accordingly, that excess hydrocortisone induced a decrease in placental explants' metabolic activity in our setting. Despite the increase in its protein level, placental glucose uptake was decreased, suggesting that the mechanism underlying this effect may well be a GC-induced translocation of glucose transporters from the plasma membrane to intracellular sites, as it is induced in fibroblasts and adipose tissue [46, 47]. The glucose concentrations used in our model are unlikely the cause for the changes observed in this glucose transporter, since there is experimental evidence showing no changes in GLUT1 levels or activity with glucose concentrations ranging from 1 to 12 mM of glucose [48]. As in the studies mentioned before, we also found an increase in GLUT3 mRNA expression. In term placentas, GLUT3 is only expressed in the endothelium of arterial sections of placental vasculature [49, 50], and it has been speculated that an increase in its levels may relate to low fetal glucose levels, in an attempt to aid in glucose extraction from maternal circulation [51]. Our data supports that the GC induced decrease in glucose uptake in our model promotes such placental adaptive response, regardless of the fetal compartment.

Since glucose is the primary fuel required for placental and fetal growth and development [5], when its uptake is compromised, the placenta could alternatively use lipid oxidation for energy generation. In our hands, term placental explants failed to compensate the decreased glucose uptake by increasing FAO. Moreover, excess hydrocortisone induced a dose-dependent decrease in FAO in our model, as it has been shown to be induced in other tissues [52]. This is in accordance with our previous findings in a different model of IUGR as it is the pregnancy complicated with preeclampsia [53]. When the placenta decreases its FAO activity, it may induce fatty acid esterification to increase lipid stores, as we have shown in placentas from pregnancies complicated with gestational diabetes [34, 54]. On the contrary, we found that hydrocortisone induced a decrease in FAE activity. The finding that placental explants cultured with GCs are not using, or storing lipids, points to the possibility of a decreased lipid uptake. Triglycerides need to be broken down to free fatty acids by placental lipases such as lipoprotein lipase (LPL), to be readily available for placental uptake [55, 56]. Indeed, GCs reduce LPL activity in peripheral fat depots [42]; hence this could be underlying the decreased placental lipid metabolism we found with GCs. This finding, in accordance with a previous report [57], contrasts with the increased in LPL mRNA expression levels in placentas from severe IUGR newborns with abnormal umbilical artery pulsatility index, in what could be speculated to be an attempt to restore the fuel availability to the fetus, although authors did not measure LPL activity [58].

GCs are a well-known cause of cellular death by apoptosis [59], and increased apoptosis has been reported in placentas form other causes of IUGR such as preeclampsia and HELLP syndrome [60]. Hence it was mandatory to check that the decrease in placental explants metabolism induced by GCs was not the result of an increased apoptosis, and we tested this by three different methods. We found no signs of increased apoptosis with the hydrocortisone concentrations used neither by TUNEL, Caspase Glo assay, nor by cleaved caspase 3 levels, as it could be expected from the dose curve we performed prior to the experiments to choose GCs concentrations. Thus the decrease in MTT observed with the highest concentration of hydrocortisone used could be explained by the global energetic shortage leading to a decreased metabolic activity of the placental explants, which would also be in accordance with the decreased levels of phosphorylated Erk found in response to GCs.

In summary, our data in placental explants suggest that GCs excess disables human term placenta to regulate nutrient transport, affecting primarily its efficiency for nutrient uptake. It hinders placental glucose and lipid uptake independently of the nutrient availability, showing the kind of response to GCs expected from peripheral tissues.

The main limitation of the study which recommends a cautious interpretation of the results is the high hydrocortisone concentrations used. Studies on glucocorticoids' effects on placenta* ex vivo* are usually performed with dexamethasone or other synthetic corticoids. We intentionally used hydrocortisone as an example of endogenous glucocorticoid; thus we had to perform preliminary studies to set the hydrocortisone concentrations in our experiments. Interestingly, we only found one article using hydrocortisone* ex vivo* in a similar tissue (umbilical cord), and it was designed to test the effects of different corticoids on fetal vasculature [61]. The range of hydrocortisone concentrations tested went from 10^−9^ to 10^-4 ^M, and the relaxant effect of hydrocortisone on the vasculature was dose dependent and peaked at the maximum concentration tested (10^-4 ^M). If the authors had tested concentrations of 10^-3 ^M (in the range of the ones used in our experiments), it is plausible to speculate on an even higher effect, suggesting that the hydrocortisone concentrations needed to exert effects in* ex vivo* models of placental tissue could be much higher than physiological concentrations.

Similarly, the resulting concentrations we finally used are relevantly higher than physiological cortisone concentrations* in vivo*, but we previously proved that they did not induce cellular apoptosis while inducing cellular metabolism changes in the placental explants, resembling well documented hydrocortisone metabolic effects on peripheral organs.

## 5. Conclusions

Excess hydrocortisone reduces human term placenta explants' ability to uptake glucose and lipids, and their ability to use these as energy source, inducing a cellular metabolic arrest. This fuel shortage to the placenta may plausibly contribute to a disrupted placental development and placental insufficiency, leading to an inefficient nutrient transport to the fetus. These findings suggest that growth restriction of fetus from mothers needing corticoid therapy during pregnancy is the result of fetal nutrient deprivation, besides corticoids' direct effect on the fetus. The fetal adaptive responses to this condition may result in developmental programming of metabolism for fuel shortage favoring the development of lifelong metabolic morbidities.

## Figures and Tables

**Table 1:** Maternal, gestational, and newborns' characteristics.

Parameter	Value
Maternal Age (years)	35.22 ± 0.74
Gestational Age (weeks)	38.79 ± 0.41
Maternal Glucose (mg/dl)	86.89 ± 3.65
Maternal Total Cholesterol (mg/dl)	175.2 ± 6.12
Maternal HDL-Chol (mg/dl)	69.56 ± 0.99
Maternal Triglycerides (mg/dl)	67.00 ± 5.13
Placental weight (g)	584.0 ± 36.58
Newborn weight (g)	3351 ± 98.01
Newborn weight (percentile)	62.56 ± 5.99

Values are expressed as means ± SEM.

## Data Availability

Complete raw datasets generated during the study are archived as IP's personal files, in Graph Pad Prism software, and are publicly available upon request to the corresponding author.

## References

[1] Barker D. J. P. (2004). The developmental origins of adult disease. *Journal of the American College of Nutrition*.

[2] Vitale G., Salvioli S., Franceschi C. (2013). Oxidative stress and the ageing endocrine system. *Nature Reviews Endocrinology*.

[3] Battaglia F. C., Meschia G. (1988). Fetal Nutrition. *Annual Review of Nutrition*.

[4] Jones H. N., Powell T. L., Jansson T. (2007). Regulation of placental nutrient transport - a review. *Placenta*.

[5] Brett K. E., Ferraro Z. M., Yockell-Lelievre J., Gruslin A., Adamo K. B. (2014). Maternal–fetal nutrient transport in pregnancy pathologies: the role of the placenta. *International Journal of Molecular Sciences*.

[6] Fowden A. L., Ward J. W., Wooding F. P. B., Forhead A. J., Constancia M. (2006). Programming placental nutrient transport capacity. *The Journal of Physiology*.

[7] Pardi G., Marconi A. M., Cetin I. (2002). Placental-fetal interrelationship in IUGR fetuses—a review. *Placenta*.

[8] Gerretsen G., Huisjes H. J., Elema J. D. (1981). Morphological changes of the spiral arteries in the placentae bed in relation to pre‐eclampsia and fetal growth retardation. *BJOG: An International Journal of Obstetrics & Gynaecology*.

[9] Khong T. Y., de Wolf F., Robertson W. B., Brosens I. (1986). Inadequate maternal vascular response to placentation in pregnancies complicated by pre-eclampsia and by small-for-gestational age infants. *British Journal of Obstetrics & Gynaecology*.

[10] Roland M. C., Friis C. M., Voldner N. (2012). Fetal growth versus birthweight: the role of placenta versus other determinants. *PLoS ONE*.

[11] Wallace J. M., Horgan G. W., Bhattacharya S. (2012). Placental weight and efficiency in relation to maternal body mass index and the risk of pregnancy complications in women delivering singleton babies. *Placenta*.

[12] Godfrey K. M. (2002). The role of the placenta in fetal programming - A review. *Placenta*.

[13] Eriksson J., Forsén T., Tuomilehto J., Osmond C., Barker D. (2000). Fetal and childhood growth and hypertension in adult life. *Hypertension*.

[14] Jansson N., Pettersson J., Haafiz A. (2006). Down-regulation of placental transport of amino acids precedes the development of intrauterine growth restriction in rats fed a low protein diet. *The Journal of Physiology*.

[15] Jansson T., Cetin I., Powell T. L. (2006). Placental Transport and Metabolism in Fetal Overgrowth - A Workshop Report. *Placenta*.

[16] Johansson M., Karlsson L., Wennergren M., Jansson T., Powell T. L. (2003). Activity and protein expression of Na+/K+ ATPase are reduced in microvillous syncytiotrophoblast plasma membranes isolated from pregnancies complicated by intrauterine growth restriction. *The Journal of Clinical Endocrinology & Metabolism*.

[17] Jansson T., Powell T. L. (2006). Human placental transport in altered fetal growth: Does the placenta function as a nutrient sensor? A review. *Placenta*.

[18] Seckl J. R., Holmes M. C. (2007). Mechanisms of disease: glucocorticoids, their placental metabolism and fetal 'programming' of adult pathophysiology. *Nature Clinical Practice Endocrinology & Metabolism*.

[19] McTernan C. L., Draper N., Nicholson H. (2001). Reduced placental 11*β*-hydroxysteroid dehydrogenase type 2 mRNA levels in human pregnancies complicated by intrauterine growth restriction: an analysis of possible mechanisms. *The Journal of Clinical Endocrinology & Metabolism*.

[20] Goland R. S., Troppe P. J., Warren W. B., Stark R. I., Jozak S. M., Conwell I. M. (1995). Concentrations of corticotrophin-releasing hormone in the umbilical-cord blood of pregnancies complicated by pre-eclampsia. *Reproduction, Fertility and Development*.

[21] Chi C.-C., Wang S.-H., Mayon-White R., Wojnarowska F. (2013). Pregnancy outcomes after maternal exposure to topical corticosteroids: A UK population-based cohort study. *JAMA Dermatology*.

[22] Murphy K. E., Willan A. R., Hannah M. E. (2012). Effect of antenatal corticosteroids on fetal growth and gestational age at birth. *Obstetrics & Gynecology*.

[23] MacArthur B. A., Howie R. N., Dezoete J. A., Elkins J. (1982). School progress and cognitive development of 6-year-old children whose mothers were treated antenatally with betamethasone. *Pediatrics*.

[24] French N. P., Hagan R., Evans S. F., Godfrey M., Newnham J. P. (1999). Repeated antenatal corticosteroids: Size at birth and subsequent development. *American Journal of Obstetrics & Gynecology*.

[25] Yeh T. F., Lin Y. J., Lin H. C. (2004). Outcomes at school age after postnatal dexamethasone therapy for lung disease of prematurity. *The New England Journal of Medicine*.

[26] Braun T., Challis J. R., Newnham J. P., Sloboda D. M. (2013). Early-life glucocorticoid exposure: The hypothalamic-pituitary-adrenal axis, placental function, and longterm disease risk. *Endocrine Reviews*.

[27] Fowden A. L., Forhead A. J., Sferruzzi-Perri A. N., Burton G. J., Vaughan O. R. (2015). Review: Endocrine regulation of placental phenotype. *Placenta*.

[28] Newnham JP., Evans SF., Godfrey M., Huang W., Ikegami M., Jobe A. (1999). Maternal, but not fetal, administration of corticosteroids restricts fetal growth. *The Journal of Maternal-Fetal & Neonatal Medicine*.

[29] Unek G., Ozmen A., Kipmen-Korgun D., Korgun E. T. (2012). Immunolocalization of PCNA, Ki67, p27 and p57 in normal and dexamethasone-induced intrauterine growth restriction placental development in rat. *Acta Histochemica*.

[30] Ozmen A., Unek G., Kipmen-Korgun D., Korgun E. T. (2011). The expression of Akt and ERK1/2 proteins decreased in dexamethasone- induced intrauterine growth restricted rat placental development. *Journal of Molecular Histology*.

[31] Turkay E., Ozmen A., Unek G., Mendilcioglu I., Qian X. (2012). The effects of glucocorticoids on fetal and placental development. *Glucocorticoids - New Recognition of Our Familiar Friend*.

[32] Kipmen-Korgun D., Ozmen A., Unek G., Simsek M., Demir R., Korgun E. T. (2012). Triamcinolone up-regulates GLUT 1 and GLUT 3 expression in cultured human placental endothelial cells. *Cell Biochemistry & Function*.

[33] Korgun E. T., Acar N., Sati L. (2011). Expression of glucocorticoid receptor and glucose transporter-1 during placental development in the diabetic rat. *Folia Histochemica et Cytobiologica*.

[34] Visiedo F., Bugatto F., Sánchez V., Cózar-Castellano I., Bartha J. L., Perdomo G. (2013). High glucose levels reduce fatty acid oxidation and increase triglyceride accumulation in human placenta. *American Journal of Physiology-Endocrinology and Metabolism*.

[35] Perdomo G., Martinez-Brocca M. A., Bhatt B. A., Brown N. F., O'Doherty R. M., Garcia-Ocaña A. (2008). Hepatocyte growth factor is a novel stimulator of glucose uptake and metabolism in skeletal muscle cells. *The Journal of Biological Chemistry*.

[36] Perdomo G., Commerford S. R., Richard A.-M. T. (2004). Increased *β*-oxidation in muscle cells enhances insulin-stimulated glucose metabolism and protects against fatty acid-induced insulin resistance despite intramyocellular lipid accumulation. *The Journal of Biological Chemistry*.

[37] Hughes S. D., Quaade C., Johnson J. H., Ferber S., Newgard C. B. (1993). Transfection of AtT-20(ins) cells with GLUT-2 but not GLUT-1 confers glucose-stimulated insulin secretion. Relationship to glucose metabolism. *The Journal of Biological Chemistry*.

[38] Brown N. F., Stefanovic-Racic M., Sipula I. J., Perdomo G. (2007). The mammalian target of rapamycin regulates lipid metabolism in primary cultures of rat hepatocytes. *Metabolism - Clinical and Experimental*.

[39] Bligh E. G., Dyer W. J. (1959). A rapid method of total lipid extraction and purification. *Canadian Journal of Physiology and Pharmacology*.

[40] Mosmann T. (1983). Rapid colorimetric assay for cellular growth and survival: application to proliferation and cytotoxicity assays. *Journal of Immunological Methods*.

[41] Saif Z., Hodyl N. A., Hobbs E. (2014). The human placenta expresses multiple glucocorticoid receptor isoforms that are altered by fetal sex, growth restriction and maternal asthma. *Placenta*.

[42] Vegiopoulos A., Herzig S. (2007). Glucocorticoids, metabolism and metabolic diseases. *Molecular and Cellular Endocrinology*.

[43] Langdown M. L., Sugden M. C. (2001). Enhanced placental GLUT1 and GLUT3 expression in dexamethasone-induced fetal growth retardation. *Molecular and Cellular Endocrinology*.

[44] Barrosa L. F., Yudilevich D. L., Jarvis S. M., Beaumont N., Baldwin S. A. (1995). Quantitation and immunolocalization of glucose transporters in the human placenta. *Placenta*.

[45] Ebert B. L., Firth J. D., Ratcliffe P. J. (1995). Hypoxia and mitochondrial inhibitors regulate expression of glucose transporter-1 via distinct cis-acting sequences. *The Journal of Biological Chemistry*.

[46] Carter-Su C., Okamoto K. (1987). Effect of insulin and glucocorticoids on glucose transporters in rat adipocytes. *American Journal of Physiology-Endocrinology and Metabolism*.

[47] Horner H. C., Munck A., Lienhard G. E. (1987). Dexamethasone causes translocation of glucose transporters from the plasma membrane to an intracellular site in human fibroblasts. *The Journal of Biological Chemistry*.

[48] Illsley N. P., Sellers M. C., Wright R. L. (1998). Glycaemic regulation of glucose transporter expression and activity in the human placenta. *Placenta*.

[49] Mouzon S. H.-d., Challier J. C., Kacemi A., Caüzac M., Malek A., Girard J. (1997). The GLUT3 glucose transporter isoform is differentially expressed within human placental cell types. *The Journal of Clinical Endocrinology & Metabolism*.

[50] Illsley N. P. (2000). Glucose transporters in the human placenta. *Placenta*.

[51] Baumann M. U., Deborde S., Illsley N. P. (2002). Placental glucose transfer and fetal growth. *Endocrine Journal*.

[52] Lettéron P., Brahimi-Bourouina N., Robin M.-A., Moreau A., Feldmann G., Pessayre D. (1997). Glucocorticoids inhibit mitochondrial matrix acyl-CoA dehydrogenases and fatty acid *β*-oxidation. *American Journal of Physiology-Gastrointestinal and Liver Physiology*.

[53] Bartha J. L., Visiedo F., Fernández-Deudero A., Bugatto F., Perdomo G. (2012). Decreased mitochondrial fatty acid oxidation in placentas from women with preeclampsia. *Placenta*.

[54] Visiedo F., Bugatto F., Quintero-Prado R., Cózar-Castellano I., Bartha J. L., Perdomo G. (2015). Glucose and fatty acid metabolism in placental explants from pregnancies complicated with gestational diabetes mellitus. *Reproductive Sciences*.

[55] King J. C. (2006). Maternal obesity, metabolism, and pregnancy outcomes. *Annual Review of Nutrition*.

[56] Duttaroy A. K. (2009). Transport of fatty acids across the human placenta: A review. *Progress in Lipid Research*.

[57] Magnusson-Olsson A. L., Hamark B., Ericsson A., Wennergren M., Jansson T., Powell T. L. (2006). Gestational and hormonal regulation of human placental lipoprotein lipase. *Journal of Lipid Research*.

[58] Tabano S., Alvino G., Antonazzo P., Grati F. R., Miozzo M., Cetin I. (2006). Placental LPL gene expression is increased in severe intrauterine growth-restricted pregnancies. *Pediatric Research*.

[59] Distelhorst C. W. (2002). Recent insights into the mechanism of glucocorticosteroid-induced apoptosis. *Cell Death & Differentiation*.

[60] Cali U., Cavkaytar S., Sirvan L., Danisman N. (2013). Placental apoptosis in preeclampsia, intrauterine growth retardation, and HELLP syndrome: an immunohistochemical study with caspase-3 and bcl-2. *Clinical and Experimental Obstetrics & Gynecology*.

[61] Potter S. M., Dennedy M. C., Morrison J. J. (2002). Corticosteroids and fetal vasculature: Effects of hydrocortisone, dexamethasone and betamethasone on human umbilical artery. *BJOG: An International Journal of Obstetrics & Gynaecology*.

